# Topiramate induced bilateral hypopyon uveitis and choroidal detachment: a report of two cases and review of literature

**DOI:** 10.1186/s12886-021-02050-x

**Published:** 2021-07-27

**Authors:** Mudit Tyagi, Shashwat Behera, Sirisha Senthil, Rajeev R. Pappuru, Vikas Ambiya, Siddharth Dikshit

**Affiliations:** 1 Uveitis and Ocular Immunology Services, Smt Kanuri Santhamma Center for Vitreo Retinal Diseases, L. V. Prasad Eye Institute, Hyderabad, 500034 India; 2 Smt Kanuri Santhamma Center for Vitreoretinal Diseases, Hyderabad, India; 3 VST Glaucoma Services L. V. Prasad Eye Institute, Hyderabad, India

**Keywords:** Topiramate, Inflammation, Choroidal detachments, Hypopyon

## Abstract

**Background:**

Topiramate (TPM) is a drug commonly used by neurophysicians and psychiatrists for a plethora of indications. Topiramate has been reported to induce acute angle closure glaucoma as an adverse effect. However, there is limited literature on Topiramate causing hypopyon uveitis and intense ocular inflammation. It is imperative for ophthalmologists as well as physicians to be aware of the potential sight threatening ocular adverse effects of Topiramate. We report 2 rare consecutive cases of severe hypopyon uveitis and choroidal detachments after using Topiramate.

**Case presentation:**

Two patients presented with sudden onset of angle closure, bilateral hypopyon uveitis and choroidal detachments. On reassessing a detailed treatment history, it was found that both patient were taking oral Topiramate which had been started 2 weeks before the onset of ocular symptoms. The bilateral hypopyon and angle closure were considered to be induced by Topiramate and the drug was discontinued. The patients were started on oral and topical steroids which led to resolution of hypopyon uveitis and choroidal detachments. The visual acuity improved and the intraocular pressure also got normalised in both the cases.

**Conclusions:**

Topiramate can lead to a bilateral hypopyon uveitis and severe ocular inflammation. An urgent cessation of topiramate along with topical and systemic steroids is required to prevent serious complications.

## Background

Topiramate (TPM) is a drug commonly used by neurophysicians and psychiatrists for several indications including epilepsy, prophylaxis for migraine, alcohol and tobacco dependence, infantile spasms, essential tremors, bipolar disorders, and obsessive- compulsive disorders [1, 2]. Topiramate has been reported to cause acute angle closure glaucoma as an adverse effect [2]. However, there is a limited literature on Topiramate causing hypopyon uveitis and intense ocular inflammation. In this case report we describe two consecutive cases in which patient developed bilateral hypopyon uveitis and choroidal detachments as an unexpected, non-dosage related adverse drug reaction after starting Topiramate.

## Case presentation

### Case 1

A 54 year old woman presented to the Uveitis Services with acute painful diminution of vision in both eyes since 10 days. She was initially suspected to have a bilateral acute angle closure glaucoma and she had undergone a surgical peripheral iridectomy in her left eye at another hospital 1 week before reporting to us. At the time of presentation, she was already using topical timolol eyedrops along with oral acetazolamide .

**Fig. 1:**
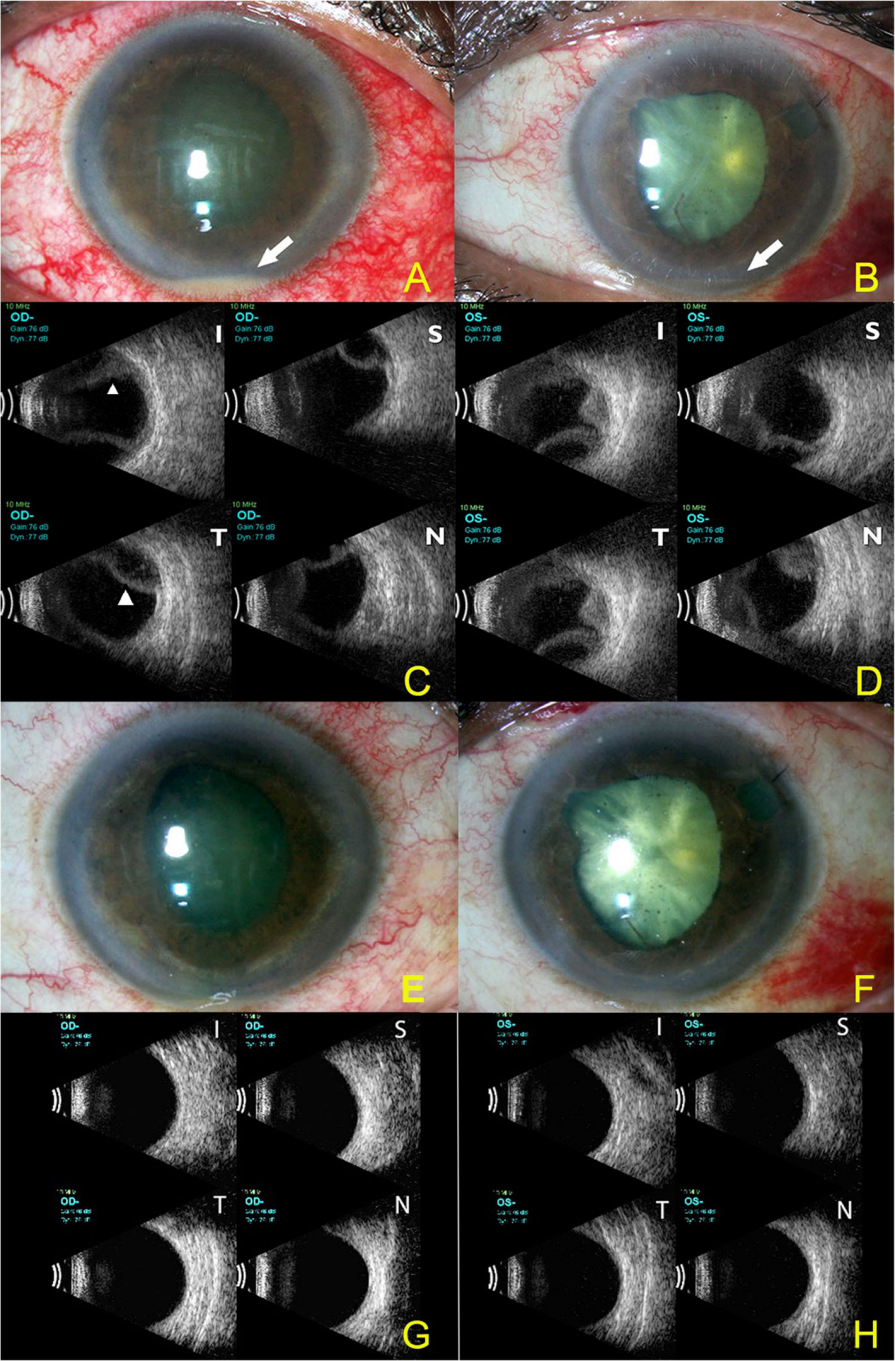
**A** & **B** Colour slit lamp photograph of the right and left eye showing hypopyon (white arrows). **C** & **D** Ultrasound B scan of the right and left eye showing choroidal detachments in all four quadrants (white arrowhead) (S: superior; I: inferior; N: Nasal; T: Temporal). The hypopyon resolved 2 weeks after stopping topiramate and starting topical and systemic steroids. **E** & **F** Colour slit lamp photograph of the right and left eye showing resolved hypopyon **G** & **H** Ultrasound B scan of the right and left eye showing resolved choroidal detachments in all quadrants

She had a distant visual acuity of light perception with inaccurate projection of rays in her right eye and counting fingers at one metre in her left eye. Both the eyes had corneal stromal edema along with flare + 2, cells+ 2 and a hypopyon approximately 1 mm in height along with fixed and dilated pupils, and cataract, (Fig. 1A & B) and no view of fundus due to hazy media. The Intraocular pressure (IOP) was 10 and 8 mm of Hg in the right and left eye respectively. An Ultrasound B scan showed choroidal detachments in all quadrants in both the eyes (Fig. 1C & D). A clinical diagnosis of bilateral panuveitis and bilateral endogenous endophthalmitis was considered. However, there was no history suggestive of any systemic source of infection as a cause for endogenous endophthalmitis and a systemic evaluation along with complete uveitic workup including chest X-ray to exclude sarcoidosis and tuberculosis, Mantoux test for tuberculosis, serology for HIV and syphilis and HLA B51 for Behcet’s disease was done which turned out to be negative.

On reassessing a detailed treatment history, it was found that the patient was suffering from depression and had been started on oral Topiramate (100 mg per day) which had been started 2 weeks before the onset of her ocular symptoms. The bilateral hypopyon and angle closure were considered to be induced by Topiramate and the drug was therefore discontinued. The patient was started on oral prednisolone (1 g/kg body weight) and prednisolone acetate eyedrops and topical cycloplegics (atropine 1% eyedrops). After 2 weeks, her visual acuity had improved to 20/200 in right eye and counting fingers at 1 m in left eye. The reaction in the anterior chamber (Fig. 1E & F) had regressed and the choroidal detachments had resolved (Fig. 1G & H). At 1 month of follow up the vision in her right eye had improved to 20/100 and was counting fingers at1 m in the left eye. The vision in left eye was less due to the cataractous changes and she was planned for cataract surgery later in the left eye.

### Case 2

**Fig. 2:**
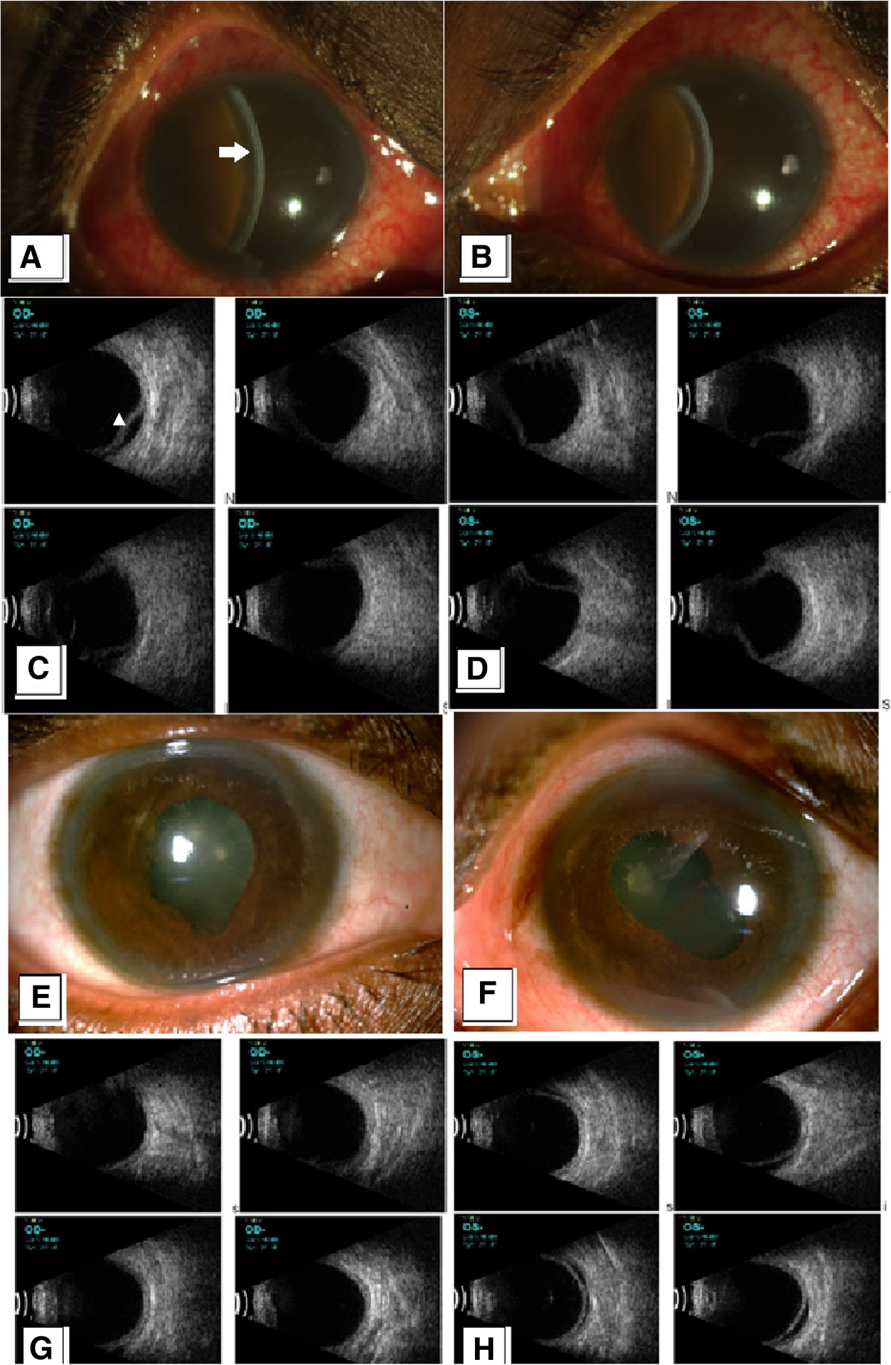
**A** & **B** Colour slit lamp photograph of the right and left eye showing corneal stromal edema with shallow anterior chamber (white arrow). **C** & **D** Ultrasound B scan of the right and left eye showing choroidal detachments in all four quadrants (arrowhead) (S: superior; I: inferior; N: Nasal; T: Temporal). The corneal edema resolved after stopping topiramate and starting the patient on topical and systemic steroids. **E** & **F** Colour slit lamp photograph of the right and left eye showing clear cornea and regressed anterior chamber inflammation at 1 month of follow up. **G** Ultrasound B scan of the right showing resolved choroidal detachments in all quadrants. **H** Ultrasound B scan of left eye showing resolving choroidal detachment. The visual acuity improved to a snellens equivalent of 20/50 in the right eye and 20/40 in the left eye

A 43-year-old woman presented to the Uveitis clinic with chief complains of sudden painful diminution of vision in both eyes since 2 days. She had a history of head trauma with associated subdural hematoma following road traffic accident 6 months earlier. On examination, both eyes had a visual acuity of light perception with inaccurate projection of rays along with circumciliary congestion, corneal stromal edema and bilateral intense anterior chamber reaction with flare + 4, cells + 4 with associated shallow anterior chamber depth and a streak hypopyon (Fig. 2A and B). The IOP was 4 mm of Hg in the right eye and 7 mm of hg in the left eye at the time of presentation. Retinal details and posterior segment details were not visible due to the corneal edema. An ultrasound B scan of both eyes revealed choroidal detachments in all four quadrants in both eyes (Fig. 2C and D). The patient gave a history of having been started on oral topiramate (100 mg daily) for her migraine 2 weeks earlier by her physician. Based on the above clinical features and a history of Topiramate exposure since 2 weeks, a diagnosis of bilateral drug induced anterior uveitis was made. She was asked to discontinue Topiramate and was started on systemic and topical corticosteroids along with topical atropine eyedrops. At 1 month of follow up, the visual acuity in her right eye had improved to 20/50 in the right eye and in the left eye it had improved to 20/40. The IOP had increased to 10 and 11 mm of Hg in the right and the left eye and there was a complete resolution of choroidal detachment at the end of 1 month. (Fig. 2E-H).

## Discussion

Topiramate (TPM) is a drug which is commonly used by neurophysicians and psychiatrists. The main indications for its use include epilepsy, prophylaxis for migraine, alcohol dependence, tobacco dependence, infantile spasms, essential tremor, bipolar disorder, and obsessive- compulsive disorder [1].

Topiramate has been reported to induce acute angle closure glaucoma as an adverse effect [2]. The mechanism for secondary close-angle glaucoma is believed to be cilio-choroidal effusion, anterior rotation of the ciliary body and a forward displacement of the iris-lens diaphragm with closure of the anterior chamber angle. However, its propensity to occasionally cause a severe ocular inflammation and uveitis associated with hypopyon is not so well known. We found few reports associating Topiramate with anterior uveitis and hypopyon uveitis and panuveitis [3–8]. Goldberg et al. had reported 7 cases of topiramate-associated uveitis after a literature search through data mining of the Food and Drug Administration Adverse Event Reporting System and cumulative review of cases from a global safety database and published literature [4]. Mahendradas et al. had reported a case of a 36-year-old lady who was taking topiramate for migraine and had developed sudden diminution of vision in both the eyes along with panuveitis and angle-closure glaucoma [5].

Both our cases presented with a sudden onset of severe ocular inflammation along with choroidal detachments. The patients initially had a bilateral angle closure followed by choroidal detachments and a severe bilateral panuveitis associated with a hypopyon. However both our cases had low IOP at time of presentation. While Case 1 had a history of already using Tab acetazolamide at time of presentation to our clinic, case 2 had presented with hypotony along with a shallow anterior chamber. The hypotony in the second case could be attributed to the ciliary body shutdown and subsequent to the choroidal detachments caused by the inflammation. After discontinuing Topiramate and initiating treatment with topical and systemic steroids, the inflammation had regressed and the IOP had also normalised along with resolution of the choroidal detachments .

Pikkel et al. had reported a case of Topiramate induced uveitis where intravenous methylprednisolone was used along with topical steroids for resolving the panuveitis [6].

Considering the severity of the adverse effect of this drug, it was not possible to re-challenge with the drug to prove its causality. We assessed the association of Topiramate with bilateral hypopyon uveitis using Naranjo’s algorithm [9] and the WHO–UMC Probability Scale and found that the drug had a probable association with the event of bilateral hypopyon uveitis in our cases. The cilio-choroidal effusion and hypopyon uveitis caused by Topiramate therefore is an idiosyncratic dose independent response. The clinical resolution of uveitis after discontinuing Topiramate suggests a causal relationship.

Occasionally even masquerade syndrome can present as bilateral hypopyon uveitis and should be considered as a differential diagnosis. However a history of Topiramate use points towards such a presentation being secondary to an idiosyncratic dose independent response to Topiramate.

Kamal et al. had reported about the cross reactivity of Topiramate with sulfonamide derivatives and therefore it is prudent to exercise caution in the use of acetazolamide in management of glaucoma secondary to Topiramate [7].

Apart from the cross sensitivity, acetazolamide in itself has been reported to cause angle closure and ciliochoroidal effusion [10].

Keeping in view, the wide-spread use of Topiramate in present times by physicians, psychiatrists, and neurologists, it is imperative for all to be aware of the potential ocular adverse effects of this drug.

In conclusion, a diagnosis of drug associated uveitis should always be considered in cases with bilateral acute choroidal detachments and bilateral hypopyon uveitis. An urgent cessation of Topiramate therapy is needed in these cases along with topical and systemic steroids to prevent serious ocular complications.

## Data Availability

All the data pertaining to the cases is provided in the manuscript and can be shared upon request.
